# Regulation of Rice Grain Quality by Exogenous Kinetin During Grain-Filling Period

**DOI:** 10.3390/plants14030358

**Published:** 2025-01-24

**Authors:** Yunhua Xiao, Yating Dong, Meng Zhou, Yingfeng Wang, Xiong Liu, Xuedan Lu, Guilian Zhang, Feng Wang, Wenbang Tang, Huabing Deng

**Affiliations:** 1 College of Agronomy, Hunan Agricultural University, Changsha 410128, China; 2 Hunan Provincial Key Laboratory of Rice and Rapeseed Breeding for Disease Resistance, Changsha 410128, China; 3 Yuelushan Laboratory, Changsha 410128, China; 4 State Key Laboratory of Hybrid Rice, Hunan Hybrid Rice Research Center, Changsha 410125, China

**Keywords:** rice (*Oryza sativa* L.), rice grain quality, cytokinin, cooked rice elongation, starch granule morphology, rice grain swelling rate

## Abstract

Cytokinins (CKs) play important functions in plant growth and development and in response to adversity stress. However, little is known about the role CK plays in rice grain quality. We hypothesized that exogenous cytokinins could improve rice grain quality by regulating physiological traits and genes related to starch synthesis. Therefore, we exogenously applied different concentrations of kinetin (KT), an exogenous CK, during the grain-filling period. Our results show that all the different concentrations of exogenous KT treatments resulted in a significant increase in thousand-grain weight. In particular, chalkiness and chalky grain rate were significantly reduced, and gel consistency (GC) content and alkali spreading value (ASV) were significantly increased in 10^−8^ M KT treatment. Meanwhile, the exogenous application of 10^−8^ M KT positively affected the transcription of some starch synthesis-related genes, which was in contrast to the 10^−5^ M KT treatment. In conclusion, the exogenous application of appropriate concentrations of KT during the grain-filling period can ultimately affect rice grain quality by regulating the changes in the relevant indicators, such as appearance quality (AQ) and eating and cooking qualities (ECQ).

## 1. Introduction

Rice (*Oryza sativa* L.) is vital to the livelihoods and diets of more than 3.5 billion people [1]. The world is rich in rice resources, and with advances in scientific research, rice production has increased dramatically and is expected to reach 567 million tons by 2030 [2]. With the continuous improvement of the economic level, people’s demand for rice grain quality is also increasing; at the same time, the production of high rice grain quality has also played a solid role in the sustainable development of the rice value chain to a certain extent [3]. Improving rice grain quality has been a top priority in rice research. Therefore, it is necessary to explore effective methods to improve rice grain quality.

The rice grain quality can be judged by the characteristics embodied in appearance quality (AQ), milling quality (MQ), eating and cooking quality (ECQ), and nutritional quality (NQ) [4]. AQ is mainly determined by grain shape and chalkiness [5]. Importantly, this influences consumer choice [6]. The evaluation of MQ is through brown rice rate (BRR), milled rice rate, and head rice rate [7]. MQ is of particular importance to farmers, as it determines to some extent the final yield and the rate of broken rice, and likewise is one of the main influences on the fluctuations in the value of the product [7]. The main features of ripe rice are ECQ [8]. ECQ can often be assessed indirectly using data obtained from amylose content (AC), gel consistency (GC), and rapid viscosity analyzer (RVA) [9]. Starch consists mainly of amylose and amylopectin [10]. AC is widely recognized as one of the most important determinants of the ECQ of rice [11]. It will affect the stickiness and hardness of the rice, resulting in a different texture of the rice [12]. Measurement of the viscosity characteristics of grain starches using RVA offers greater reliability, reproducibility, and functionality [13].

External factors are one of the main factors affecting rice grain quality, including temperature [14,15], light [16], fertilizer application [17], sowing period [18], density [19], water management [20], etc. Elevated expression of *Amy1A*, *Amy3C*, and *Amy3D* in the endosperm of ripening seeds at high temperatures is the main cause of chalky grain production [21]. Not only that, but high temperatures also reduce seed weight and thousand-grain weight [22]. On the contrary, the fertilizer application at the spike stage improves chalkiness and effectively mitigates the poor rice grain quality [23]. Adopting an efficient moisture management approach alternative wetting and drying (AWD) can improve milling recovery and seed quality [20]. In addition, rice grain quality is also influenced by genetic factors, and this influence is complex, with individual traits potentially being controlled by multiple genes, as well as traits interacting with each other. *OsPIL15* negatively regulates seed size, thousand-grain weight, and chalkiness by modulating cytokinin content [24]. *Wx*, *Wx^a^*, *Wx^b^*, and *Wx^in^* alleles can significantly affect the softness and texture of rice by regulating AC [25]. *ALK* is a key gene for gelatinization temperature (GT), and when GT rises, it leads to a decrease in peak viscosity, which further has an effect on the paste ability of rice [26].

Cytokinins (CKs) are a very important phytohormone that is involved in plant growth and developmental processes, as well as stress-related responses [27]. It not only contributes to cell division and differentiation but also down aging and protects against adversity stresses [28]. By mutating rice CK OXIDASE/DEHYDROGENASE3 (OsCKX3), CK content at the lamina joint was altered and upregulated, resulting in reduced leaf angle [29]. *OsNAC2* can mediate root growth and development by regulating crosstalk between auxin and CK [30]. In addition, numerous studies have shown that CKs are closely related to grain size, grain weight, and yield. *TaCKX6-D1* haplotype expression variation is closely related to seed weight and thousand-grain weight [31]. *RGG1* regulates rice seed size by affecting the content of CK [32]. External spraying of CK during the grain-filling period can realize the purpose of improving rice yield [33].

Kinetin (KT) is the best-known CK and is often used as an exogenous plant regulator due to its favorable synthetic properties. Kinetin was the first CK, isolated in 1955 [34,35,36,37]. CKs are important phytohormones in plants, which play an important role in regulating plant growth processes and enhancing plant responses to biotic and adversity stresses, among which the cytokinin KT has a greater potential for development because of its very superior properties [38]. Exogenous application of KT increased the tolerance of rice seedlings to high temperatures [39] and can also be used to achieve stress tolerance under abiotic stresses by directly delaying leaf senescence [40]. Under drought conditions, growth- and yield-related traits can even be enhanced by exogenous application of KT to reduce the deleterious effects [41]. It was also shown that foliar spraying of tomato seedlings with KT alleviated UV-B stress [42]. Spraying KT on the leaves of corn plants is very effective in reducing the toxicity of boron and provides good protection [43]. Unfortunately, there is still a relative gap in research on KT in improving rice grain quality, which needs to be further expanded and explored. We hypothesized that when in the grain-filling period, exogenous cytokinins could regulate the AQ and ECQ. Therefore, we investigated the possibility of exogenously applying KT to improve rice-grain-quality-related characteristics. The conclusion of this study will give us further insight into the link between KT and rice grain quality.

## 2. Materials and Methods

### 2.1. Plant Material and Experimental Design

The indica early rice cultivar Zhongzao39 was used for the study. Rice plants were placed in culture chambers for hydroponic growth. Growing conditions: 28 °C during the day, 28 °C at night, 12 h day/night, 70% humidity, 30,000 Lux light intensity. Nutrition was provided to seedlings by using Kimura B nutrient solution (pH 5.6–5.8). Rice seedlings at 10 days of age were removed from the incubator, placed outdoors for two days, and then transplanted into potting buckets containing planting soil with good nutrient conditions. Before the rice plants grew to the spike stage, they were transferred to an artificial climate chamber and incubated under the same conditions as light intensity and humidity. After the 10th day of heading, 500 μL kinetin (KT) with 10^−10^, 10^−9^, 10^−8^, 10^−7^, 10^−6^, and 10^−5^ M concentrations, respectively, were dripped from top to bottom along the rachis with a pipette gun at 10:00 a.m. each day for continuous 30 days treatment. Treatment with 0 M KT concentration served as the control. Seeds were harvested 7 days after the end of the treatment, and the relevant indicators were determined.

### 2.2. Determination of Milling Quality and Grain Phenotyping of Rice Grain

Grains were harvested and threshed, dried naturally, and stored for 3 months. Referring to the national standard GB/T 24535-2009 (https://std.samr.gov.cn/, the same as below), the length and width of the grains were determined by Rice Appearance Analysis System (SC-E, Wseen, Hangzhou, China), with the thickness measured by vernier calipers [44]. Determination of brown rice rate (BRR) was performed according to agricultural industry standard of China, NY/T 83-1988. The milled rice rate was determined according to the agricultural industry standard of China, NY/T 147-1988. Determination of the head rice rate was carried out according to the national standard GB/T 21719-2008.

### 2.3. Determination of Chalkiness and Chalky Grain Rate

Rice grains from each treatment were harvested and processed into milled rice. Chalkiness and chalky rice rate were examined using a chalkiness observation instrument (SC-E, Wseen, Hangzhou, China) [45].

### 2.4. Determination of the Moisture Content, Amylose Content (AC), Gel Consistency (GC), and the Alkali Spreading Value (ASV)

The weight of a single aluminum box is denoted as M_1_ (g). The actual total weight of the box and sample before drying is denoted as M_2_ (g), after drying the actual total weight of the box and sample is denoted as M_3_ (g). After obtaining the actual data for M_1_, M_2_, and M_3,_ the data are brought into the formula for calculation. Sample moisture content (%) = (M_2_ − M_3_/M_2_ − M_1_) × 100%. As for the determination of amylose content (AC), the relevant test operation is carried out according to the national standard GB/T 15683-2008, and the samples and standard samples were digested overnight after being wetted with anhydrous ethanol and kept at 37 °C ambient temperature in sodium hydroxide solution. Then, after adding glacial acetic acid and potassium iodide solution to make it color developed, the OD value was measured at 620 nm. The AC of the test samples was further calculated from the standard curve performed by the AC in the standard samples [46].

As for the gel consistency (GC), with reference to the national standard GB/T 22294-2008, ethanol phenol blue indicator and potassium hydroxide solution were added to the test tubes containing rice flour and mixed by thorough shaking. The reaction was carried out in boiling water, at room temperature, and in an ice-water bath after placing an appropriate glass ball in the mouth of the test tube; final horizontal resting time is 1 h. The straight-line distance from the bottom of the glass test tube to the tip of the fully solidified rice glue is considered the GC value [47].

For the determination of alkali spreading value (ASV), we referred to the Chinese agricultural industry standard NY/T 147-1988. Intact particles without cracks and of uniform size were selected and immersed in a solution of KOH (0.304 M, 10 mL) at a constant temperature of 30 °C, from which they were immersed for 23 h. The diffusion cracking degree was mainly used as the basis for grading each rice grain [48].

### 2.5. Determination of Cooked Rice Elongation and Swelling Rate

Head rice grains were selected, and their length and width were measured with vernier calipers. Place the above head rice in a test tube, 7.5 mL distilled water was added, placed in boiling water for 30 min, and then steamed for 10 min. Finally, the head rice was taken out and dried on filter paper at room temperature for 3 h, and then the length and width of the rice were measured. The ratio of cooked rice length to head rice length was measured as cooked rice elongation, and the ratio of cooked rice width to head rice width as the swelling rate.

### 2.6. Determination of Rice Starch Viscosity

Rapid viscosity analyzer (RVA) testing refers to the national standard GB/T 24852-2010. These include peak viscosity (PKV), trough viscosity (TV), cool paste viscosity (CPV), breakdown viscosity (BDV), recovery values (RVs), setback viscosity (SBV). Analysis performed with the rapid visco analyzer (Newport Scientific Pty, Warriewood, NSW, Australia) [49].

### 2.7. Scanning Electron Microscopy (SEM)

The cross sections of milled rice were observed under scanning electron microscopy (JSM-6380LV) with magnification of ×35, ×100, and ×500, respectively.

### 2.8. Quantitative Real-Time PCR

Rice grains were harvested after 4 h of treatment with 10^−5^ M and 10^−8^ M KT on the 15th day of continuous treatment. The total RNA extraction, reverse transcription, and qRT–PCR assays were performed as described previously [50]. The rice *Ubiquitin2* was used as the internal reference gene and for the normalization in the analyses. Throughout the experiment, three biological replicates were performed. Primers used for qRT–PCR are listed in Table S1.

### 2.9. Experimental Design and Statistical Analysis

During the artificial climate outdoor source KT experiment, the same potting soil was used in the potting buckets. Three buckets were set up for each concentration with six plants per bucket and three tillers per treatment. The original data were statistically measured by Microsoft Excel, and the mean value and standard deviation were calculated. Different letters in the figures and tables indicate significant differences (*p* < 0.05) derived from one-way ANOVA and least significant difference (LSD) test. GraphPad Prism (v8.0.2) was used for plotting.

## 3. Results and Discussion

### 3.1. Granulation

**Figure 1:**
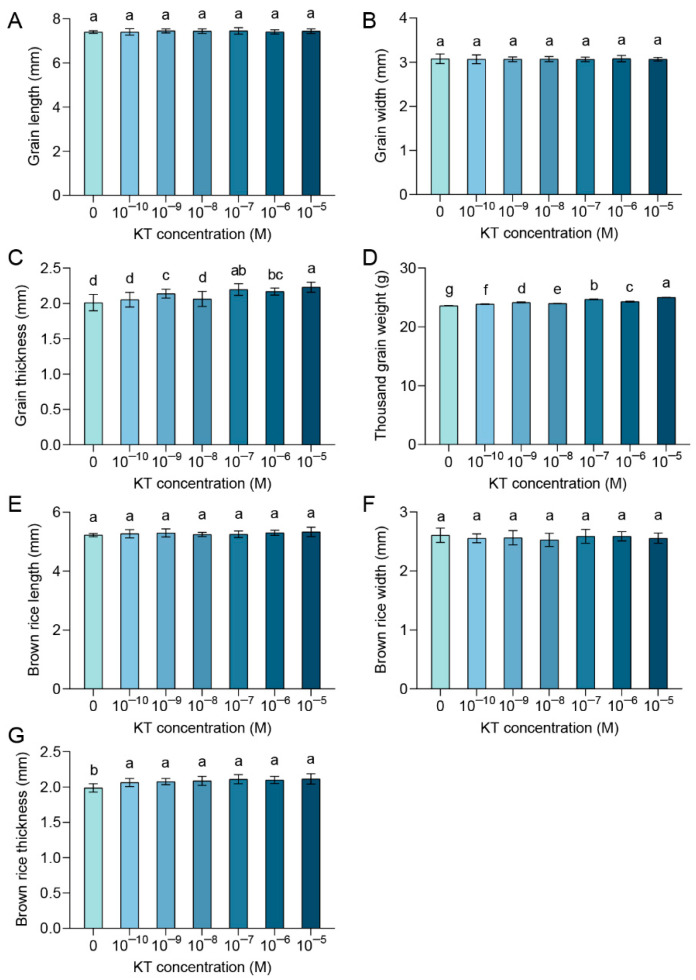
Exogenous application of 10^−8^ M KT can increase thousand-grain weight by increasing the brown rice thickness: (**A**–**D**) Influence of different concentrations of KT treatments on grain length (**A**) and grain width (**B**), grain thickness (**C**), and thousand grain weight (**D**). (**E**–**G**) Changes in brown rice length (**E**), brown rice width (**F**), and brown rice thickness (**G**) under different KT concentration treatments. Values are means ± standard deviation. Different lowercase letters in the graphs indicate significant differences at the *p* < 0.05 level, grain length *n* = 20, grain width *n* = 20, grain thickness *n* = 20, thousand-grain weight *n* = 3, brown rice length *n* = 10, brown rice width *n* = 10, brown rice thickness *n* = 10.

With the different concentrations of kinetin (KT) treatments, we measured grain length, grain width, grain thickness, and thousand-grain weight of Zhongzao39. The results show that different concentrations of KT treatments had little effect on grain length and width (Figure 1A,B). This may be related to the timing of the treatments, at about 10 days of heading, the development of the husk is complete, and the phenotype of grain length and width is already in a relatively fixed state. Grain thickness was increased by 6%, 9%, 8%, and 11% in 10^−9^, 10^−7^, 10^−6,^ and 10^−5^ M KT treatments, respectively, compared with the control (Figure 1C). This suggests that the various concentrations of KT treatments led to an increase in grain fullness to some extent, a phenomenon that was particularly evident at the higher concentrations. It is noteworthy that all concentrations of KT treatment led to a highly significant increase in thousand-grain weight over the control (Figure 1D). Compared with the control, the largest increase in thousand-grain weight was observed at 10^−5^ M KT treatments (6%, *p* < 0.05), and the smallest increase in thousand-grain weight was observed at 10^−10^ M KT treatments (1%, *p* < 0.05). This means that exogenously applied KT in our study had the same yield-increasing effect as other exogenous KT treatments [51], which has a positive impact on actual production.

To further investigate the effect of exogenous KT treatment on brown rice, we measured the length, width, and thickness of brown rice. All concentrations of KT treatments showed no significant effect on the length and width of brown rice compared with the control (Figure 1E,F). Notably, all concentrations of KT treatments resulted in a highly significant increase in brown rice grain thickness (Figure 1G). This result suggests that exogenous cytokinin (CK) treatment further increases the thousand-grain weight mainly by increasing the grain thickness of brown rice. It has been shown that KT possesses the property of causing cell division [52], and the grain fullness can be further improved by applying exogenous CK [53]. Therefore, exogenously applied KT has the potential to promote cell division during the grain-filling period, leading to an increase in grain thickness, which further leads to an increase in thousand-grain weight.

### 3.2. Appearance Quality and Starch Granule Morphology

**Figure 2:**
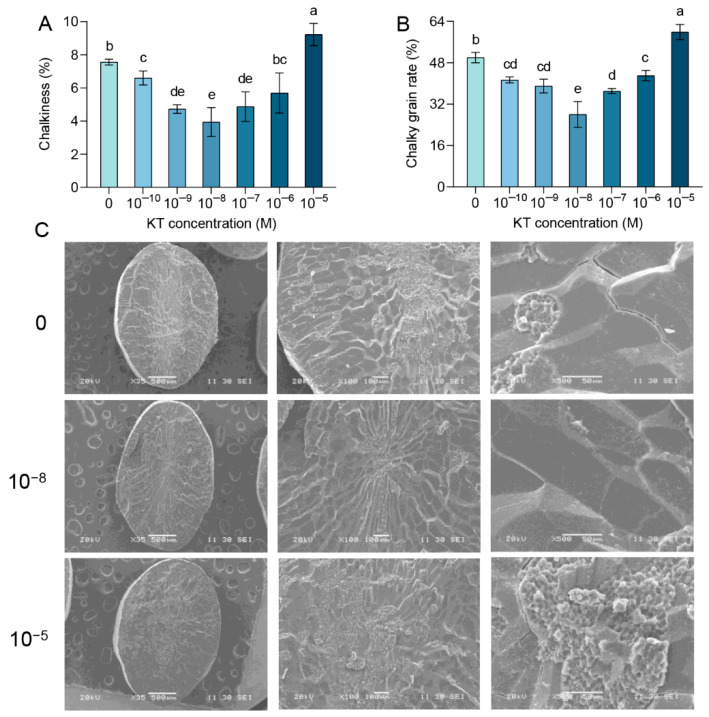
Exogenous application of 10^−8^ M KT was effective in reducing chalkiness and chalky grain rate: (**A**, **B**) Impact of different concentrations of KT treatments on chalkiness (**A**) and chalky grain rate (**B**). (**C**) Results of scanning electron microscopy under 10^−8^ and 10^−5^ M KT treatments. Values are means ± standard deviation. Different lowercase letters in the graphs indicate significant differences at the *p* < 0.05 level, *n* = 3.

Chalkiness is an important indicator for assessing the rice grain quality [54]. The opaque regions within the endosperm are a defining characteristic of chalkiness, arising from the loose arrangement of starch granules [55]. This configuration increases light scattering, thereby adversely affecting the visual quality of rice. At the same time, high chalkiness can cause the rice to break easily during milling, which further reduces the milling rate and head rice rate [56]. Consequently, this phenomenon not only diminishes the luster of the grains but also potentially lowers their market value and consumer acceptance [57]. As can be seen in Figure 2A,B, the chalkiness of the 10^−10^, 10^−9^, 10^−8^, and 10^−7^ M KT treatments decreased to varying degrees compared with the control. Similarly, not only was the chalky grain rate reduced under these four concentration treatments as compared with the control, but the same reduction was also observed in the 10^−6^ M KT treatment. The greatest reductions in chalkiness and chalky grain rate were achieved in the 10^−8^ M concentration treatment, with 48% and 44%, respectively. On the contrary, both chalkiness and chalky grain rates were significantly increased by 22% and 20% in the 10^−5^ M KT treatment relative to the control. The most critical period with rice grain quality is the second week after spike emergence, which is crucial for the adequacy of endosperm cell development and grain filling [58]. This suggests that the application of appropriate concentrations of CK at the right time may reduce chalkiness by regulating grain filling.

With a horizontal slice of rice identified with SEM, we hoped to explain the variation in chalkiness by the density and void structure of the starch arrangement in the rice. The morphology and size distribution of starch granules have the greatest influence on chalky grain rate [59], and their internal structure and physicochemical properties are mainly due to genetics and the external environment [9]. Irregular morphology and non-uniform distribution of starch granules can lead to reduced light transmission, which can exacerbate chalkiness and chalky grain rate [60]. The chalkiness and chalky grain rate compared with the control were most pronounced in the 10^−8^ M and 10^−5^ M KT concentration treatments, so these two treatments were selected for observation (Figure 2A,B). The SEM results reveal that under 10^−8^ M KT treatment, the starch granules in rice are densely packed, with cells exhibiting a regular and full shape, as compared with the control (Figure 2C). This phenomenon suggests that 10^−8^ M KT treatment promotes the compact arrangement of starch granules, thereby reducing chalkiness. Upon careful examination of the electron microscopy images, it is evident that under 10^−5^ M KT treatment, the central lamellar starch structures exhibit a radiating pattern compared with the group, with an increase in the number of fissures. Additionally, multiple protrusions aggregate to form cluster-like structures, resulting in noticeable voids between the starch granules (Figure 2C). This is consistent with the significant increase in chalkiness and chalky grain rate in the 10^−5^ M KT treatment. Plant hormone interactions and complex downstream regulatory networks play an important role in the formation of chalkiness [61]. It has been shown that knockout of the *OsCKXs* gene affects chalky grain rate [62]. Our results show that exogenous application of different concentrations of KT affected the alignment density and granule morphology of starch granules, and exogenous KT at 10^−8^ M was the most effective in reducing chalkiness. Conversely, research findings indicate that high concentrations of cytokinin (KT) may exacerbate the degree of chalkiness. This may be due to the effect of exogenous CK on the CK regulatory network, resulting in changes in grain-filling rate. Therefore, selecting the appropriate concentration of cytokinin (CK) and applying it at the proper time during the rice growth process can effectively mitigate the chalkiness phenomenon.

### 3.3. Milling Quality

Brown rice rate (BRR), milled rice rate, and head rice rate are usually used as an important basis for assessing the quality of milled rice [63]. In order to study the effect of KT treatment on the milled rice quality, we measured and analyzed those three indices of the grains harvested after different concentrations of KT treatment (Table 1). The results show that 10^−10^ M KT treatment significantly reduced the BRR by 2% compared with the control, while other concentration treatments had little effect on it. Milled rice rate was significantly reduced by 3% and 2% under further 10^−9^ M KT and 10^−6^ M KT treatments, respectively, compared with the control. In addition, we found that all concentrations (10^−10^, 10^−9^, 10^−8^, 10^−7^, 10^−6^, 10^−5^) of exogenous KT treatments resulted in varying decreases in head rice rate compared with controls, with reductions of 14%, 19%, 19%, 12%, 17%, and 11%, respectively. Reduced starch, increased chalkiness, or abnormal moisture content can cause a reduction in head rice yield [64]. Rice with a low head rice rate tends to result in increased broken rice, which reduces its utility and economic value [65]. These results indicate that different concentrations of exogenous KT treatment had an adverse effect on the milling quality, reducing the quality of rice. It provides important guidance for future studies on the effects of KT on rice quality.

### 3.4. Amylose Content, Gel Consistency, and Alkali Spreading Value

**Figure 3:**
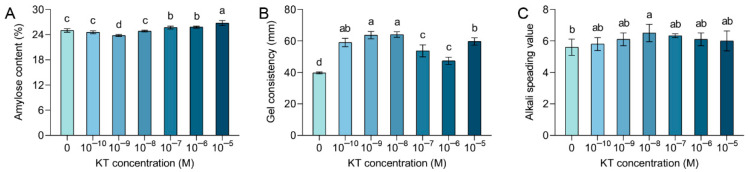
Effect of different KT concentration treatments on amylose content, gel consistency, and alkali spreading value. Content changes in amylose content (**A**), amylose content (**B**), and alkali spreading value (**C**) after treatment with different concentrations of KT. Values are means ± standard deviation. Different lowercase letters in the graphs indicate significant differences at the *p* < 0.05 level, with AC and GC *n* = 3, and ASV *n* = 6.

In order to investigate the effect of exogenous KT on cooking flavor, we examined the contents of amylose content (AC), gel consistency (GC), and alkali spreading value (ASV). AC is an important indicator for assessing the cooking flavor, textural, and nutritional qualities of rice [4]. As shown in Figure 3A, the application of 10^−9^ M KT treatment significantly declined AC content by 5% as compared with the control. During the exogenous KT treatment, it was observed that the AC content in the 10^−10^ and 10^−8^ M KT treatment groups showed no significant difference compared with the control group. However, with the escalation of KT concentration to 10^−7^, 10^−6^, and 10^−5^ M, there was a marked increase in AC levels. The AC content significantly increased by 3%, 3%, and 7% when KT concentration was increased to 10^−7^, 10^−6^, and 10^−5^ M, respectively, as compared with the control (Figure 3A). The cooked rice grains may become softer as the AC content decreases. Thus, from the results, it is evident that exogenously applied KT at a concentration of 10^−9^ M made the cooked semolina softer, thereby increasing its palatability and better meeting the preferences of some of the consumers concerned [66].

The properties of AC are often described by their GC, and it is generally recognized that a gel consistency between 41 mm and 60 mm is medium hard, less than 40 mm is hard, and more than 60 mm is soft [4]. All concentrations of KT treatments resulted in a significant increase in GC compared with the control GC value of 39.6 mm, with the 10^−9^ and 10^−8^ M treatments having the greatest effect, reaching GC values of 63.7 mm and 64 mm. At a treatment concentration of 10^−6^ M, the GC value was 47.3 mm, which was less variable relative to other concentrations. (Figure 3B). This suggests that exogenous addition of KT at concentrations of 10^−9^ M or 10^−8^ M improves GC and results in softer and more delicious cooked rice.

Cooking time of rice is also one of the concerns, it is closely related to gelatinization temperature (GT). It is common knowledge that ASV and GT are inverse indicators of each other [48], high ASV leads to low GT, resulting in shorter cooking time and softer cooked rice [67]. There were no differences in ASV results for the 10^−10^, 10^−9^, 10^−7^, 10^−6^, and 10^−5^ M concentration treatments compared with the control (Figure 3C). However, the ASV was significantly higher by 16% (*p* < 0.05) in the 10^−8^ M KT concentration treatment compared with the control; this may result in lower GT values and softer cooked rice after treatment at this concentration (Figure 3C). Based on the above results, we conclude that the exogenous application of appropriate concentrations of KT could significantly adjust AC and GC and affect the softness and palatability of cooked rice, which was particularly maximized by the 10^−9^ M KT treatment. ASV may be less susceptible to exogenous hormones, but a specific concentration of 10^−8^ M KT significantly reduced cooking time and similarly improved the softness and palatability of cooked rice compared with controls.

### 3.5. Significance Analysis of Cooked Rice Elongation

Cooked rice elongation and swelling rate are some of the indicators of rice grain quality [68]. We further investigated the effect of exogenously applied KT on cooked rice elongation. From Table 2, it is evident that the application of varying concentrations of KT exogenously can enhance the cooked rice length and elongation to different extents. Compared with the control, the concentration 10^−5^ M KT significantly increased the head rice length by 7% (*p* < 0.05), while the other concentrations had no significant effect on head rice length. Changes in head rice width were insensitive to all different concentrations of KT treatments. There was a significant increase in the cooked rice length in all the different concentrations of KT treatments compared with the control. The results for cooked rice width show that only 10^−10^ and 10^−9^ M KT treatments caused its increase. We focused on the cooked rice elongation and swelling rate. The measurement of the cooked rice elongation KT concentration could significantly increase the cooked rice elongation by 21%, 16%, 12%, 16%, 13%, and 10%, respectively, compared with the control. When compared with the control, the 10^−10^ M KT treatment significantly increased the swelling rate by 13%, whereas there was no significant change in the other concentration treatments. Higher cooked rice elongation of grains of rice is favored by more consumers [69]. Comprehensive research data show that exogenous treatment with 10^−10^ M KT and 10^−9^ M KT concentrations has greater potential application value, improving eating and cooking quality (ECQ).

### 3.6. Rapid Viscosity Determination of Starch by RVA Analysis

Rice starch pasting characteristics were measured using an operational and reproducible RVA [70]. Rapid viscosity determination of starch by RVA analysis includes peak viscosity (PKV), trough viscosity (TV), cool paste viscosity (CPV), breakdown viscosity (BDV), recovery values (RVs), setback viscosity (SBV). PKV is a maximally viscous pasteurized starch reached when heated in water [13], reflecting the solubility and water absorption of starch granules, the main constituent of rice [71]. The BDV value has been utilized to assess the ease of disintegration of swollen starch granules; a decrease in its value results in starch granules that are more difficult to disintegrate [72]. Short-term regeneration processes in rice play a major role in influencing rice hardness, and SBV can be used as an indicator of starch retrogradation [73]. Rice with a low SBV does not retrograde when cooled, which means it has good cooking qualities [74]. Tasteful rice is characterized by high PKV and BDV values and also by a low SBV value [49,75].

**Figure 4:**
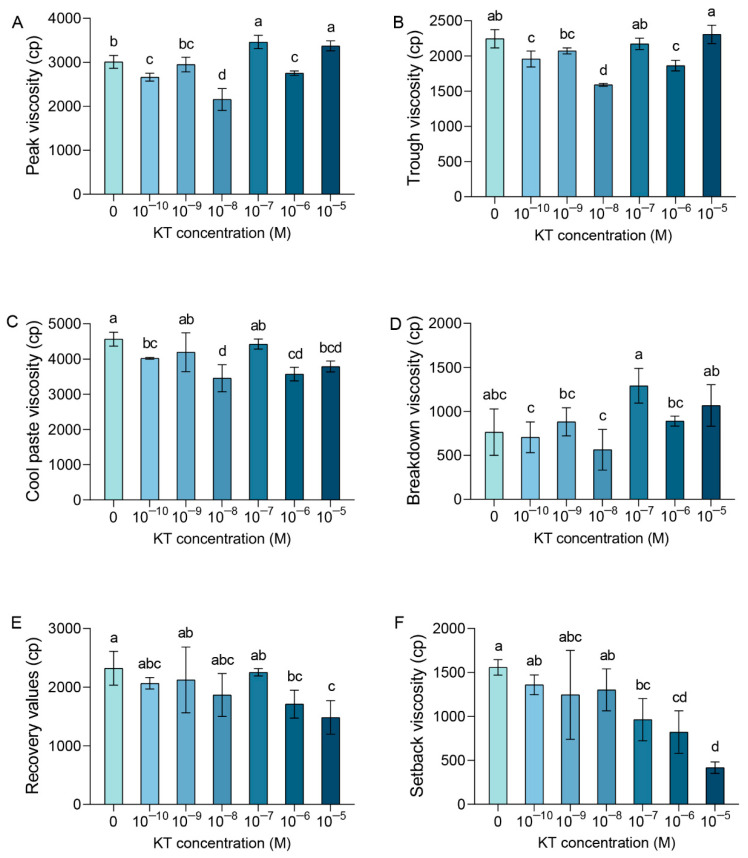
Effect of different KT concentrations treatments on RVA. Changes in peak viscosity (**A**), trough viscosity (**B**), cool paste viscosity (**C**), breakdown viscosity (**D**), recovery values (**E**), and setback viscosity (**F**) in rice grain after treatment with different concentrations of KT. Values are means ± standard deviation. Different lowercase letters in the graphs indicate significant differences at the *p* < 0.05 level, *n* = 3.

As can be seen from Figure 4, the PKV values for the 10^−7^ M and 10^−5^ M KT treatments exhibited significant increases of 15% and 12%, respectively, in comparison with the control group. On the contrary, the PKV values of 10^−10^ M, 10^−8^ M, and 10^−6^ M treatments were significantly reduced by 12%, 28%, and 9%, respectively, compared with the control as the basis. TV was significantly lower than controls at 10^−10^, 10^−8^, and 10^−6^ M KT treatments, and conversely, there was no significant difference after 10^−9^, 10^−7^, and 10^−5^ M KT treatments. Compared with the control group, the application of KT at concentrations of 10^−10^ M, 10^−8^ M, 10^−6^ M, and 10^−5^ M significantly reduced CPV values (Figure 4C). Among these, the 10^−8^ M KT treatment group demonstrated the greatest reduction in CPV of 24% compared with the control group. There was no significant difference in BDV values for different concentrations of exogenous KT treatments compared with the control (Figure 4D). The 10^−5^ M KT treatment resulted in the relatively greatest decrease in RVs compared with the control, with a 36% decrease. In addition, the 10^−6^ M KT treatment also resulted in a significant decrease of 26% (*p* < 0.05) in RVs compared with the control (Figure 4E). SBV results show that SBV was significantly reduced in 10^−7^, 10^−6^, and 10^−5^ M KT treatments compared with the control with reductions of 38%, 47%, and 73%, respectively (Figure 4F). There are a number of high-quality rice products that are used to make rice flour. The high PKV, CPV, and SBV characteristics reflect the high-quality ductility of the rice production process, which further improves the utilization of the production, as well as the chewiness of the texture [76]. Application of exogenous KT led to a decrease in RVs and SBV, which indicated an increase in the viscoelasticity of cooked rice and a decrease in the ability of rice to regenerate after the cooked rice had cooled. By analyzing the results of the RVA assays for each of the indicators, it was found that the application of exogenous KT treatments did have an effect on rice viscosity. These results combined with the previous data provide a valuable reference for future practical applications, where specific KT concentrations can be selected as needed to target the improvement of the corresponding rice quality and achieve the practical implications in production.

### 3.7. 10^−8^ M KT Treatment Increases the Transcription Level of Starch Synthesis-Related Genes

**Figure 5:**
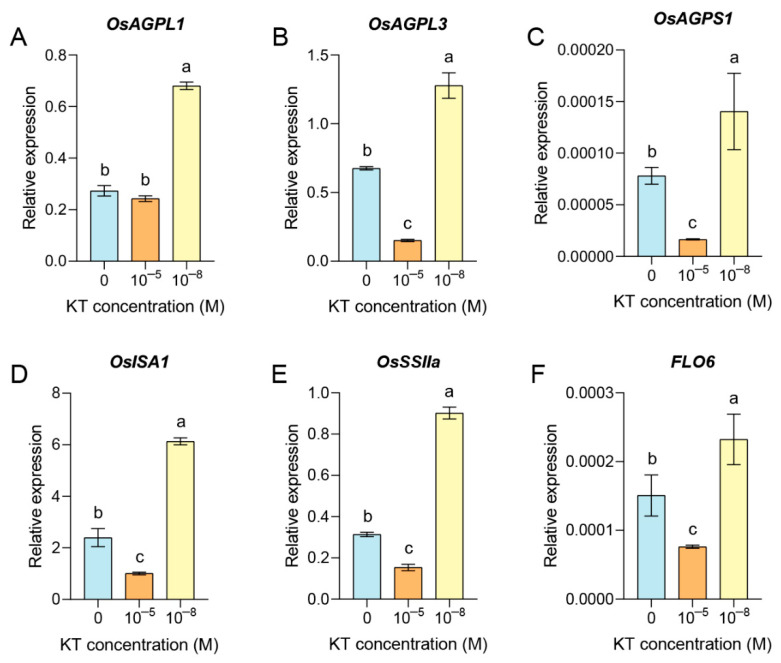
Exogenous application of 10^−8^ M KT positively affects the transcription of some starch synthesis-related genes: (*OsAGPL1*; (**A**), *OsAGPL3*; (**B**), *OsAGPS1*; (**C**), *OSISA1*; (**D**), *OsSSIIa*; (**E**), and *OsFLO6*; (**F**) in rice grain treated with 0, 10^−5^ M and 10^−8^ M KT concentrations. Data are expressed as mean ± SD. Different letters indicate significant differences by one-way ANOVA test (*n* = 3, *p* < 0.05).

In order to further analyze the possible molecular mechanisms by which exogenously applied KT affects the rice grain quality, we detected and analyzed starch synthesis-related genes using quantitative real-time PCR (qRT−PCR). These genes included *OsAGPL1*, *OsAGPL3*, *OsAGPS1*, *OsISA1*, *OsSSIIa*, and *FLO6*. As depicted in Figure 5, the expression of these starch synthesis-related genes was significantly increased after 10^−8^ M KT treatment compared with the control. In contrast, 10^−5^ M KT treatment significantly downregulated the transcript levels of these genes (except for *OsAGPL1*) compared with the control. ADP–glucose pyrophosphorylase (AGPase) plays a very critical catalytic role in plant starch biosynthesis [77]. *OsAGPL1* plays an important function in starch synthesis in culm during embryo development, also contributing to starch accumulation in the endosperm [78]. *OsAGPL1* further catalyzes starch synthesis by interacting with *OsAGPS1* [79]. It has been shown that changes in sucrose and ABA concentrations can have an effect on *OsAPL3* expression levels and starch content in cultured cells [77]. Abnormal starch granules and amyloplasts, GC were significantly elevated in *isa1* mutant endosperm cells [80]. During rice seed development, *FLO6*, a starch-binding protein, can directly interact with *OsISA1* and is equally involved in starch synthesis and starch complex granule formation [81]. *OsSSIIa* was mainly expressed in the endosperm, and RNAi repressed revealed that it had a great effect on the development of rice seeds. *OsSSIIa*/*OsSSIIIa* double repression lines showed obvious chalkiness characteristics [82]. Meanwhile, the chalkiness of the rice grains was higher under 10^−5^ M KT treatment, whereas the chalkiness of the grains was lower under 10^−8^ M KT treatment, and the appearance and neatness of the starch grains were better (Figure 2). In summary, these data suggested that exogenous application of 10^−8^ M KT may improve the rice grain quality by upregulating the expression of starch synthesis-related genes, which in turn regulates the chalkiness of the grain, as well as the appearance and arrangement of starch grains.

## 4. Conclusions

This study systematically analyzed and compared the effects of dripping different concentrations of exogenous cytokinin (CK) kinetin (KT) on the rice grain quality of Zhongzao 39 through the following five aspects: Primarily, in terms of granulation, under different concentrations of KT treatments compared with the control, none of the brown rice width and brown rice length changed, and all of the brown rice thickness increased significantly. This outcome suggests that exogenous CK treatment further increased the thousand-grain weight mainly by increasing the brown rice thickness, hence the use of exogenous KT, which increased the thousand-grain weight. Secondly, in terms of appearance quality, treatments with 10^−10^, 10^−9^, 10^−8^, 10^−7^, 10^−6^ M KT concentrations reduced chalkiness and chalky grain rate to varying degrees compared with the control, with the 10^−8^ M KT treatment leading to the greatest relative reduction in chalkiness and chalky grain rate, and only the 10^−5^ M KT treatment leading to an increase in chalkiness and chalky grain rate. The third point, in terms of milling quality, is that the application of different concentrations of KT significantly reduced the head rice rate compared with the control. Fourth, 10^−10^ M KT treatment increased cooked rice elongation and swelling rate extremely significantly, 10^−9^ M KT treatment decreased amylose content (AC), and 10^−8^ KT caused a significant increase in GC and ASV as compared with the control. Fifth, the transcript levels of starch synthesis-related genes (except for *OsAGPL1*) in rice grains were significantly reduced, and the morphology of starch grains became looser under a high concentration of 10^−5^ M KT treatment compared with the control. Differently, under the low concentration of 10^−8^ M KT, the transcript levels of starch synthesis-related genes in rice grains would be significantly increased compared with the control, and the starch grains were arranged tightly and regularly. These indicators may further lead to different eating and cooking qualities (ECQ) and will have the same effect on cooking time. Finally, in terms of starch pasting characteristics, different concentrations of KT treatments affect the relevant indexes, further leading to different softness of cooked rice.

The use of exogenous KT to improve rice grain quality in crops is very promising. Exogenous KT further improves rice yield by affecting grain filling, providing new ideas for realizing the real value of production. As for the closely related indicators, they were improved and enhanced by selecting appropriate concentrations of exogenous KT treatments to further guide practical production applications. However, how to combine this with smart agriculture to achieve efficient exogenous KT treatment and improve the rice grain quality in actual production needs further expansion and research.

## Figures and Tables

**Table 1:** Exogenous application of different KT concentration treatments had a significant negative effect on head rice rate. Values are means ± standard deviation. Different lowercase letters in the graphs indicate significant differences at the *p* < 0.05 level, *n* = 3.

KT Concentration (M)	Brown Rice Rate (%)	Milled Rice Rate (%)	Head Rice Rate (%)
0	84.8 ± 0.2 a	75.7 ± 0.4 ab	62.2 ± 0.3 a
10^−10^	83.2 ± 0.7 b	74.4 ± 0.9 bc	53.3 ± 0.7 c
10^−9^	83.9 ± 0.3 ab	73.4 ± 0.4 c	50.2 ± 0.3 e
10^−8^	84.9 ± 0.4 a	75.9 ± 1.1 ab	50.1 ± 0.7 e
10^−7^	84.0 ± 0.4 ab	76.9 ± 0.5 a	54.7 ± 0.4 b
10^−6^	84.3 ± 0.8 ab	74.1 ± 0.4 c	51.8 ± 0.3 d
10^−5^	84.9 ± 0.9 a	74.8 ± 0.1 bc	55.3 ± 0.1 b

**Table 2:** Significant analysis of cooked rice elongation after treatment with different concentrations of KT. Values are means ± standard deviation. Different lowercase letters in the graphs indicate significant differences at the *p* < 0.05 level, *n* = 10.

KT Concentration (M)	Head Rice Length (mm)	Head Rice Width (mm)	Cooked Rice Length (mm)	Cooked Rice Width (mm)	Cooked Rice Elongation (%)	Swelling Rate (%)
0	5.10 ± 0.16 b	2.58 ± 0.16 a	6.45 ± 0.43 c	2.90 ± 0.26 bc	1.27 ± 0.08 d	1.12 ± 0.07 bc
10^−10^	5.17 ± 0.21 b	2.60 ± 0.22 a	7.89 ± 0.45 a	3.28 ± 0.24 a	1.53 ± 0.08 a	1.27 ± 0.13 a
10^−9^	5.25 ± 0.26 b	2.67 ± 0.09 a	7.68 ± 0.53 ab	3.21 ± 0.24 a	1.46 ± 0.06 ab	1.20 ± 0.07 ab
10^−8^	5.22 ± 0.21 b	2.67 ± 0.13 a	7.36 ± 0.31 b	3.07 ± 0.25 abc	1.41 ± 0.07 bc	1.15 ± 0.08 bc
10^−7^	5.18 ± 0.11b	2.66 ± 0.13 a	7.63 ± 0.50 ab	3.12 ± 0.24 ab	1.47 ± 0.10 ab	1.17 ± 0.06 bc
10^−6^	5.26 ± 0.16 b	2.63 ± 0.08 a	7.50 ± 0.37 ab	2.88 ± 0.22 c	1.43 ± 0.05 bc	1.09 ± 0.06 c
10^−5^	5.46 ± 0.26 a	2.61 ± 0.26 a	7.56 ± 0.40 ab	3.09 ± 0.19 abc	1.39 ± 0.06 c	1.19 ± 0.07 b

## Data Availability

The original contributions presented in the study are included in the article; further inquiries can be directed to the corresponding author.

## Supplementary Materials

The following supporting information can be downloaded at https://www.mdpi.com/article/10.3390/plants14030358/s1, Table S1. Primers used for qRT–PCR.
