# Supplementary material for: Regulation of Rice Grain Quality by Exogenous Kinetin During Grain-Filling Period
Source: Plants (Basel). 2025 Jan 24;14(3):358. doi: 10.3390/plants14030358 (PMC11820766; doi:10.3390/plants14030358)
Supplement: Supplementary file 1 [file plants-14-00358-s001.zip › plants-3418731-supplementary.pdf]

Supplementary Table S1. Primers used for Quantitative Real-Time PCR in this study.

Primer Name	Primer sequence (5' to 3')
OsAGPL1F	GGAAGACGGATGATCGAGAAAG
OsAGPL1R	CACATGAGATGCACCAACGA
OsAGPL3F	AAGCCAGCCATGACCATTTG
OsAGPL3R	CACACGGTAGATTCACGAGACAA
OsAGPS1F	GTGCCACTTAAAGGCACCATT
OsAGPS1R	CCCACATTTCAGACACGGTTT
OsISA1F	TGCTCAGCTACTCCTCCATCATC
OsISA1R	AGGACCGCACAACTTCAACATA
OsSSIIaF	GCTTCCGGTTTGTGTGTTCA
OsSSIIaR	CTTAATACTCCCTCAACTCCACCAT
FLO6F	GCTTTAATGGGTCAGAGTGG
FLO6R	AATGATTTCCTGCTGTTAGTTG
